# Nutritional and inflammatory indices in gestational and pregestational diabetes: emphasis on the Controlling Nutritional Status (CONUT) score

**DOI:** 10.1186/s12884-025-08516-w

**Published:** 2025-11-22

**Authors:** Mustafa Rasit Ozler, Erkan Saglam, Serenat Yalcin, Ebubekir Siddik Yilmaz, Nuray Nerez

**Affiliations:** 1 Department of Obstetrics and Gynecology, Division of Perinatology, , Bursa City Hospital, Bursa, Turkey; 2 Department of Obstetrics and Gynecology, Perinatology Unit, Faculty of Medicine, Uludag University, Bursa, Nilüfer 16100 Turkey

**Keywords:** Gestational diabetes, Pregestational diabetes, CONUT score, Inflammatory indices, Pregnancy outcomes, Perinatal prognosis

## Abstract

**Objective:**

To comparatively evaluate the prognostic value of nutritional and inflammatory indices in gestational diabetes mellitus (GDM) and pregestational diabetes mellitus (PGDM), and to determine their association with adverse perinatal outcomes.

**Methods:**

This retrospective study included 280 pregnant women followed at a tertiary care center between January 2020 and December 2024. Participants were categorized into three groups: GDM (*n* = 90), PGDM (*n* = 80), and healthy controls (*n* = 110). Nutritional status was assessed using the Controlling Nutritional Status (CONUT) and Prognostic Nutritional Index (PNI) scores. Inflammatory indices including NLR, SII, SIRI, PIV, and others were calculated. Perinatal outcomes such as preterm birth, NICU admission, and Composite Adverse Perinatal Outcomes (CAPO) were recorded. ROC analysis was used to determine the predictive power of the biomarkers.

**Results:**

The PGDM group demonstrated significantly higher CONUT scores and lower PNI scores compared to the other groups (*p* < 0.001). Among inflammatory markers, NLR, SII, SIRI, and PIV were significantly elevated in PGDM cases. ROC analyses revealed that the CONUT score had strong discriminative ability in differentiating diabetic (GDM and PGDM) from non-diabetic pregnancies (AUC = 0.787; specificity = 89.1%), while the PNI score showed limited discriminative capacity (AUC = 0.291). Inflammatory indices also demonstrated moderate predictive value, particularly among PGDM cases. Higher CONUT and inflammatory scores were associated with increased rates of preterm birth, NICU admission, and CAPO (*p* < 0.001).

**Conclusion:**

Nutritional and inflammatory disturbances are more prominent in PGDM than in GDM. The CONUT score and selected inflammatory indices may serve as valuable tools for early risk stratification in diabetic pregnancies. Integration of these parameters into clinical decision-making may improve perinatal outcomes, particularly in high-risk groups such as PGDM.

## Introduction

Gestational diabetes mellitus (GDM) and pregestational diabetes mellitus (PGDM) are two of the most common metabolic disorders encountered during pregnancy and are associated with both short- and long-term adverse outcomes for both the mother and the fetus [1]. GDM is defined as glucose intolerance diagnosed for the first time during pregnancy, while PGDM refers to type 1 or type 2 diabetes that existed before pregnancy [2]. Both conditions are associated with an increased risk of preeclampsia, macrosomia, neonatal hypoglycemia, and cesarean delivery rates [3].

In recent years, a significant increase in the frequency of GDM has been observed. This increase is directly related to the rise in obesity rates, the increasing age of mothers, and the prevalence of sedentary lifestyles. According to reported data from 2017, one in seven live births was associated with GDM [4]. However, the diagnostic criteria and screening approaches for GDM vary significantly between countries. Some countries implement universal screening, while others only evaluate women with risk factors [5]. This situation reduces the comparability of prevalence estimates and presents a significant barrier to the global standardization of GDM management [6].

There are also differences in the diagnostic processes. In the single-step approach, all pregnant women undergo a 75 g oral glucose tolerance test (OGTT), while in the two-step approach, screening is first done with a 50 g glucose load, followed by diagnosis with a 100 g OGTT. However, it is still unclear which approach is superior in terms of clinical outcomes [4]. The World Health Organization (WHO) and the American Diabetes Association (ADA) recommend distinguishing between GDM and diabetes based on the degree of diabetes diagnosed during pregnancy. In this context, the updated classification in 2013 recommended distinguishing between GDM and diabetes based on glucose levels [1, 2].

In individuals with diabetes, malnutrition and systemic inflammation are commonly observed and are closely associated with perinatal outcomes. In this context, the CONUT (Controlling Nutritional Status) score, used to assess nutritional status, along with various inflammatory indices (such as neutrophil-to-lymphocyte ratio (NLR), platelet-to-lymphocyte ratio (PLR), systemic immune-inflammation index (SII), and Prognostic Nutritional Index (PNI)) are used as prognostic markers in many clinical situations. The CONUT score is an easily applicable tool in clinical settings due to its basis on simple parameters such as serum albumin, total cholesterol, and lymphocyte count [7].

Recent studies have shown that the CONUT score may also be valuable in predicting obstetric complications. For example, in pregnant women diagnosed with hyperemesis gravidarum, a significant relationship was found between the CONUT score and the need for hospitalization and the duration of hospitalization [8]. In chronic diseases such as heart failure and malignancy, it has been reported that CONUT and similar indices can predict long-term mortality [7, 9]. However, the prognostic value of these scores in pregnancy-related diabetes conditions such as GDM and PGDM has not yet been clarified [10].

Therefore, this study aims to conduct a comparative analysis of the CONUT score and inflammatory indices in pregnant women diagnosed with GDM and PGDM. Thus, the clinical differences and potential prognostic values of these parameters associated with diabetes subtypes will be revealed. Unlike prior studies that evaluated inflammatory or nutritional indices separately, the present study provides an integrated assessment of both dimensions in pregnancies complicated by gestational and pregestational diabetes.

## Materials and methods

This retrospective study was conducted at a tertiary care training and research hospital. Data from pregnant women aged 18 to 45 years who were followed at the institution between January 2020 and December 2024 were reviewed. Ethical approval was obtained from the local ethics committee (Approval Date: 16.04.2025, Decision No: 2025-8/15). Data were collected through the hospital information management system and patient records. This study was conducted in accordance with the principles of the Declaration of Helsinki on the Ethical Principles of Medical Research.

A total of 280 pregnant women who met the inclusion criteria were enrolled. Inclusion required continuous antenatal follow-up at the study center from the first trimester to delivery, availability of laboratory data from 7 to 14 weeks of gestation, a 75-g oral glucose tolerance test (OGTT) performed between 24 and 28 weeks, and delivery at the same institution. All laboratory parameters used for the calculation of CONUT, PNI, and inflammatory indices (NLR, SII, SIRI, PIV, MII-1–3) were obtained from blood samples collected at 7–14 weeks of gestation during the first antenatal visit. This timing was intentionally selected to ensure that all measurements reflected early-pregnancy status before major physiological changes in hematologic or metabolic parameters occurred. According to the World Health Organization criteria, women with at least one abnormal value on the OGTT were assigned to the GDM group (*n* = 90) [1]. Pregnant women with a confirmed diagnosis of diabetes mellitus prior to conception, regardless of type (Type 1 or Type 2), were classified as having pregestational diabetes mellitus (PGDM) (*n* = 80). Diagnosis was based on preconceptional medical records, documented use of antidiabetic medication (insulin or oral agents), or elevated fasting plasma glucose (≥ 126 mg/dL) or HbA1c (≥ 6.5%) levels in accordance with the American Diabetes Association (ADA) criteria. Only those who had received regular endocrinological follow-up prior to and during pregnancy and who continued medical management (either insulin therapy or oral antidiabetic agents) during gestation were included in the PGDM group. Women with new-onset diabetes first diagnosed during pregnancy, those without preconceptional documentation, or cases with unclear glycemic history were excluded from the PGDM classification to ensure diagnostic accuracy and homogeneity of the study population. Additionally, a healthy control group was formed from pregnant women without any medical comorbidities (*n* = 110).

Exclusion criteria were as follows: chronic hypertension, rheumatologic or chronic inflammatory disease, suspected or confirmed malignancy, infection within two weeks prior to initial blood testing, known chronic viral infections, multiple gestation, and ultrasonographic or genetic fetal anomalies.

The following maternal variables were evaluated: age, gravida, family history of diabetes, BMI, mode of delivery, fasting blood glucose, hemoglobin, white blood cell count and subtypes, C-reactive protein (CRP), albumin, TSH, AST, ALT, HDL, LDL, triglycerides, total cholesterol, CONUT score, Prognostic Nutritional Index (PNI), and inflammatory indices including NLR, PLR, MLR, SII, SIRI, PIV, MII-1, MII-2, and MII-3. Inflammatory indices were calculated as follows: NLR = neutrophil/lymphocyte; PLR = platelet/lymphocyte; MLR = monocyte/lymphocyte; SII = (neutrophil × platelet)/lymphocyte; SIRI = (neutrophil × monocyte)/lymphocyte; PIV = (neutrophil × platelet × monocyte)/lymphocyte; MII-1 = NLR × CRP; MII-2 = PLR × CRP; MII-3 = SII × CRP. The CONUT score was calculated based on serum albumin, total cholesterol, and lymphocyte count, and interpreted as normal (0–2), mild (3–4), moderate (5–8), or severe (9–12) malnutrition. The PNI was calculated using the formula: 10 × serum albumin (g/dL) + 0.005 × lymphocyte count (/mm³).

In terms of neonatal outcomes, birth weight, gestational age at delivery, 1 st and 5th minute APGAR scores, neonatal intensive care unit (NICU) admission rates, and composite adverse perinatal outcome (CAPO) were assessed. CAPO was defined as the presence of at least one of the following: polyhydramnios, APGAR score ≤ 5 at 1 or 5 min, preterm premature rupture of membranes, preterm birth, NICU admission, or development of hypertensive disorders during pregnancy.

All GDM and PGDM cases diagnosed and managed between January 2020 and January 2025 were retrospectively included in the study. Additionally, a post hoc power analysis was conducted to assess the study’s ability to detect intergroup differences in CONUT scores. Based on a moderate effect size (Cohen’s f = 0.32), a significance level of α = 0.05, and a total sample size of 280 participants, the calculated statistical power (1–β) was 0.91. Since this value exceeds the commonly accepted threshold of 0.80 for adequate power in clinical research, the sample size was deemed sufficient to ensure robust comparisons.

Statistical analysis was performed using IBM SPSS Statistics version 26.0 (Armonk, NY, USA), and a* p*-value of < 0.05 was considered statistically significant. Numerical data were presented as mean ± standard deviation or median (interquartile range), and categorical data were expressed as percentages. Normality of data distribution was assessed using the Kolmogorov–Smirnov or Shapiro–Wilk test. Group comparisons for continuous variables were performed using one-way ANOVA (with Tukey HSD post-hoc) if normally distributed, or Kruskal–Wallis test (with Dunn’s post-hoc) if not. Categorical variables were analyzed using the chi-square test or Fisher’s exact test when appropriate.

## Results

A total of 280 pregnant women were included in the study and categorized into three groups: GDM (*n* = 90), PGDM (*n* = 80), and healthy controls (*n* = 110). The groups showed statistically significant differences in several demographic and clinical parameters. Women in the PGDM group were significantly older compared to the GDM and control groups (*p* < 0.001), and had the highest BMI values (*p* < 0.001). A family history of diabetes was most common among PGDM cases (60%) compared to GDM (40%) and controls (16.4%) (*p* < 0.001). Gestational age at delivery was significantly lower in the PGDM group (median 37 weeks), while controls delivered at later gestational weeks (median 39 weeks; *p* < 0.001). Birth weight was also significantly higher in the PGDM group (*p* = 0.002) (Table 1).

**Table 1:** Comparison of Demographic, Clinical, and birth outcome characteristics among the study groups

Variable	GDM (*n* = 90)	PGDM (*n* = 80)	Control (*n* = 110)	*p*-value
Age(years)*	29 (27–32)^a^	35 (33–37)^b^	30 (28–31)^a^	***< 0.001*** ^***1***^
Gravida*	2 (1–2)	2 (1–2)	1 (1–2)	0.1983^1^
BMI(kg/m2)*	30 (28.2–32.4)^b^	32.55 (28.38–35.97)^b^	25.4 (24.45–26.4)^a^	***< 0.001*** ^***1***^
Family History of Diabetes n (%)	36 (40%)	48 (60%)	18 (16.4%)	***< 0.001*** ^***2***^
Gestational age at delivery (week)*	38 (37–39)^b^	37 (36–38)^a^	39 (38–39)^c^	***< 0.001*** ^***1***^
Birth weight (gram)*	3500 (3350–3695)^b^	3645 (3188–3863)^c^	3300 (3150-3550^a^	***0.0021*** ^***1***^
Apgar 1. Min*	9 (8–9)	8 (7–9)	9 (9–9)	***0.018*** ^***1***^
Apgar 5. Min*	10 (9–10)	10 (8–10)	10 (10–10)	***0.011*** ^***1***^
Delivery Type *n* (%)				0.645^2^
Vaginal Delivery	44 (48.9)	36 (45)	60 (54.5)	
Cesarean Section	46 (51.1)	44 (55)	50 (45.5)	
Preterm delivery < 37 week *n* (%)	18 (20)	30 (37.5)	6 (5.5)	***< 0.001*** ^***2***^
NICU Admission *n* (%)	14 (15.6)	26 (32.5)	8 (7.3)	***0.0053*** ^***2***^
CAPO *n* (%)	20 (22.2)	36 (45)	12 (10.9)	***< 0.001*** ^***2***^

*BMI* Body Mass Index, *APGAR* Appearance, Pulse, Grimace, Activity and Respiration, *NICU* Neonatal Intensive Care Unit, *CAPO* Composite Adverse Perinatal Outcome, *IQR* Interquartile Range

Data are presented as mean number (%, percentage), or median (*IQR: Interquartile Range), where appropriate. *p* < 0.05 was considered statistically significant. ^1^ Kruskal-Wallis ^2^ Chi-Square

c > b > a Post-hoc Dunn t3 (Kruskal-Wallis)

Preterm delivery was observed in 37.5% of PGDM cases, significantly higher than in the GDM (20%) and control (5.5%) groups (*p* < 0.001). Similarly, NICU admission and composite adverse perinatal outcome (CAPO) rates were highest in the PGDM group (32.5% and 45%, respectively), followed by GDM (15.6% and 22.2%) and controls (7.3% and 10.9%) (*p* = 0.005 and *p* < 0.001, respectively) (Table 1).

Furthermore, a statistically significant difference was observed in 1 st and 5th minute APGAR scores among the groups. The PGDM group exhibited the lowest median APGAR scores at both time points [1st min: 8 (7–9); 5th min: 9 (8–10), followed by the GDM group [1st min: 9 (8–9); 5th min: 10 (9–10), while the control group had the highest scores with minimal variability [1st min: 9 (9–9); 5th min: 10 (10–10)] (*p* = 0.018 and *p* = 0.011, respectively). These findings are consistent with the increased incidence of preterm delivery and NICU admissions observed in the PGDM group and may reflect impaired neonatal adaptation due to underlying metabolic and inflammatory disturbances (Table 1).

In terms of laboratory parameters, fasting blood glucose (FBG) levels were highest in the PGDM group, followed by GDM and controls (*p* < 0.001). WBC and neutrophil counts were significantly elevated in both GDM and PGDM groups compared to controls (*p* = 0.002 and *p* < 0.001, respectively), while albumin levels were significantly lower in the PGDM and GDM groups than in controls (*p* < 0.001). Total cholesterol and triglyceride levels also differed significantly between groups (*p* < 0.001) (Table 2).

**Table 2:** Comparison of laboratory parameters among the study groups

Variable	GDM (*n* = 90)	PGDM (*n* = 80)	Control (*n* = 110)	*p*-value
FBG(mg/dl) ^¶^	88.89 ± 5.87^b^	94.58 ± 6.31^c^	83.49 ± 4.71^a^	***< 0.001*** ^***1***^
AST(IU/L)*	16 (14–19)	15 (14–19)	14 (12–20)	0.1713^2^
ALT (IU/L)*	16 (15–20)	17 (15–20)	15 (14–18)	0.0755^2^
TSH (mU/mL)*	1.2 (0.8–2.1)	1.1 (0.82–1.85)	1.1 (0.82–2.05)	0.8036^2^
Hemoglobin(g/dl)*	11.2 (10.9–12.3)	11.4 (10.8–12.02.8.02)	11.5 (11.0–12.2.0.2)	0.7267^2^
WBC(*10^3^/mm^3^) ^¶^	7.55 ± 1.39^b^	7.76 ± 1.46^b^	6.91 ± 0.85^a^	***0.0024*** ^***1***^
Neutrophil(*10^3^/mm^3^)*	5.40 (4.23–5.85)^b^	5.38 (4.40–6.22)^b^	4.45 (3.81–4.85)^a^	***0.0002*** ^***2***^
Lymphocyte(*10^3^/mm^3^)*	1.62(1.25–1.88)	1.57 (1.31–1.79)	1.53 (1.35–1.78)	0.6726^2^
Monocyte(*10^3^/mm^3^)*	0.49 (0.47–0.62)	0.51 (0.50–0.61)	0.49 (0.44–0.63)	0.1517^2^
Platelet(*10^3^/mm^3^)*	216 (199–265)	200 (185–254)	199 (186–225)	0.0577^2^
CRP(mg/L)*	4.8 (3.7–5.5)	5.25 (3.82–6.25)	4.5 (3.95–6.1)	0.51^2^
Triglyceride (mg/dL)*	165 (155–180)^a^	163 (136–180)^a^	180 (174–182)^b^	***< 0.001*** ^***2***^
HDL (mg/dL)*	52 (48–55)	52 (48–56)	52 (48–55)	0.5588^2^
LDL (mg/dL)*	104 (94–108)	104 (97–112)	104 (98–110)	0.8693^2^
Total Cholesterol(mg/dl)*	165 (150–182)^a^	181 (170–190)^b^	160 (133–179)^a^	***< 0.001*** ^***2***^
Albumin (g/dL) ^¶^	3.15 ± 0.33^a^	3.04 ± 0.38^a^	3.35 ± 0.27^b^	***< 0.001*** ^***1***^

*FBG* Fasting Blood Glucose AST: Aspartate transaminase, *ALT* Alanine transaminase, *TSH* Thyroid Stimulating Hormone, *WBC* White blood count, *CRP* C-Reactive Protein, *HDL* High-Density Lipoprotein, *LDL* Low-Density Lipoprotein

Data are presented as mean mean ± standard deviation (^¶^) or median (*IQR: Interquartile Range), where appropriate. *p* < 0.05 was considered statistically significant. 1: ANOVA test 2: Kruskal-Wallis

c > b > a Post-hoc Tukey (ANOVA)

c > b > a Post-hoc Dunn t3 (Kruskal-Wallis)

Evaluation of nutritional and inflammatory indices revealed that the total CONUT score was significantly higher in PGDM patients [median: 5 (4–6), followed by GDM [3 (3,–5), and was lowest in controls [3 (2–4) (*p* < 0.001). The proportion of patients with moderate malnutrition according to the CONUT classification was markedly higher in PGDM (82.5%) compared to GDM (46.7%) and none in the control group (*p* < 0.001). The PNI score was significantly lower in the PGDM group compared to controls (*p* < 0.001) (Table 3).

**Table 3:** Comparison of nutritional and inflammatory indices among the study groups

Variable	GDM (*n* = 90)	PGDM (*n* = 80)	Control (*n* = 110)	*p*-value
Total CONUT Score*	3 (3–5)^b^	5 (4–6)^c^	3 (2–4)^a^	***< 0.001*** ^***1***^
CONUT Category normal *n* (%)	8 (8.9)^a^	0 (0)^a^	108 (98.2)^b^	***< 0.001*** ^***2***^
CONUT Category mild *n* (%)	40 (44.4)^b^	6 (7.5)^a^	2 (1.8)^a^	***< 0.001*** ^***2***^
CONUT Category moderate *n* (%)	42 (46.7)^b^	66 (82.5)^c^	0 (0)^a^	***< 0.001*** ^***2***^
CONUT Category severe *n* (%)	0 (0)	8 (10)	0 (0)	***< 0.001*** ^***2***^
PNI Score^¶^	39.97 ± 4.23	38.22 ± 4.38^a^	41.41 ± 3.3^b^	***< 0.001*** ^***3***^
Neutrophil to lymphocyte ratio (NLR)*	2.92 (2.27–4.1)	3.36 (2.6–4.16)^b^	2.77 (2.29–3.29)^a^	***0.031*** ^***1***^
Platelet to lymphocyte ratio (PLR)*	139.8 (116.1–176.0)	126.8 (106.9–176.0)	134.2 (110.3–154.9.3.9)	0.6315^1^
Monocyte to lymphocyte ratio (MLR)*	0.34 (0.26–0.40)	0.33 (0.31–0.4)	0.33 (0.27–0.39)	0.4891^1^
Systemic immune inflammation index (SII)*	650.1 (485.9–1015.5.9.5)	721.0 (525.8–896.2.8.2)^b^	542.9 (448.8–703.5.8.5)^a^	***0.0154*** ^***1***^
Systemic inflammation response index (SIRI)*	1.52 (1.12–2.01)	1.88 (1.45–2.23)^b^	1.38 (1.1–1.89)^a^	***0.0138*** ^***1***^
Pan-immune inflammation value (PIV)*	350.2 (250.1–474.5.1.5)	388.6 (290.4–546.1.4.1)^b^	298.2 (220.8–381.2.8.2)^a^	***0.0114*** ^***1***^
Multi Inflammatory Index-1 (MII-1)*	14.2 (10.5–21.7)	17.6 (11.4–26.1)	12.2 (9.8–19.3)	0.0602^1^
Multi Inflammatory Index-2 (MII-2)*	685.2 (497.0–862.6.0.6)	776.6 (469.7–982.9.7.9)	644.2 (455.9–888.0)	0.5133^1^
Multi Inflammatory Index-3 (MII-3)*	2974.1 (2197.1–4606.3.1.3)	3762.9 (2151.4–5500.8.4.8)	2677.1 (1779.3–3778.5.3.5)	***0.0432*** ^***1***^

*CONUT* Controlling Nutritional Status, *PNI* Prognostic Nutritional Index

Data are presented as mean median (*IQR: Interquartile Range), (^¶^), number (%, percentage) or mean ± standard deviation where appropriate. *p* < 0.05 was considered statistically significant. 1: Kruskal-Wallis 2: Chi-square 3: ANOVA test

c > b > a Post-hoc Dunn t3 (Kruskal-Wallis)

c > b > a Post-hoc Tukey (ANOVA)

Among inflammatory indices, NLR, SII, SIRI, and PIV values were significantly higher in PGDM compared to controls (*p*-values ranging from 0.01 to 0.03), while PLR, MLR, and MII-1 to MII-3 showed no statistically significant differences across all groups, with the exception of MII-3 which reached borderline significance (*p* = 0.043) (Table 3).

**Fig. 1:**
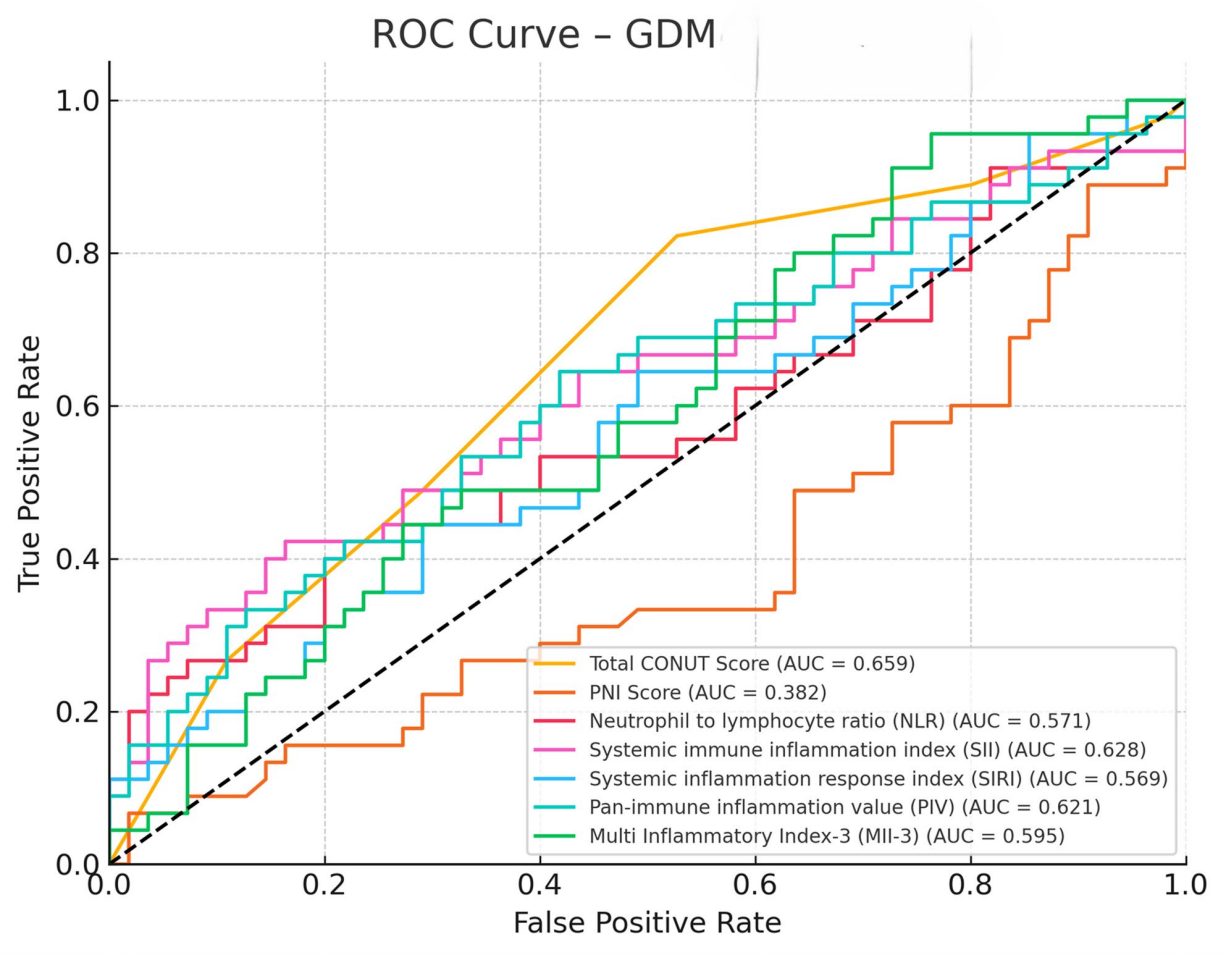
ROC curves showing the discriminative performance of CONUT, PNI, and inflammatory indices (NLR, SII, SIRI, PIV, MII-3) in distinguishing GDM from healthy pregnancies

**Fig. 2:**
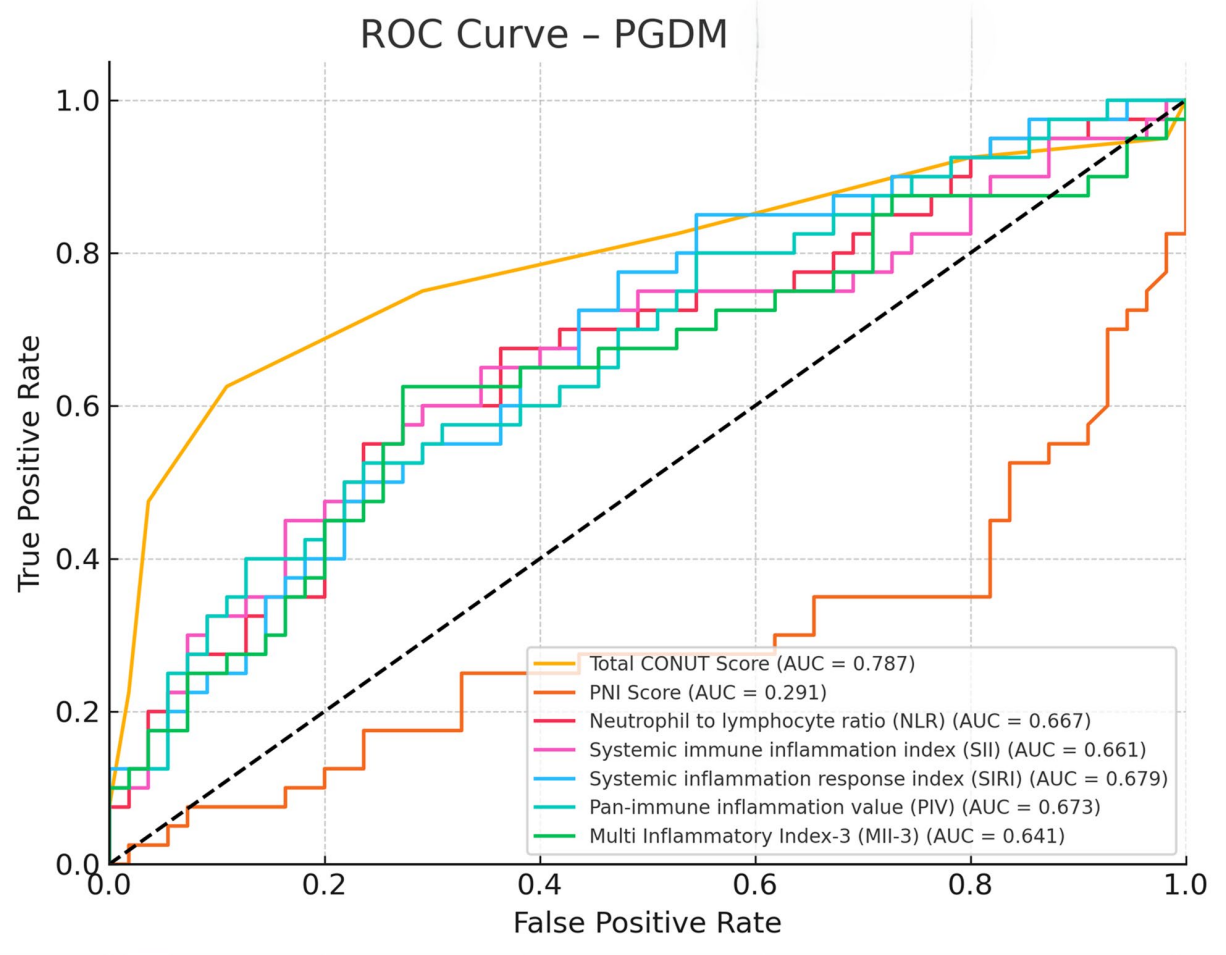
ROC curves showing the discriminative performance of CONUT, PNI, and inflammatory indices (NLR, SII, SIRI, PIV, MII-3) in distinguishing PGDM from healthy pregnancies

ROC curve analyses demonstrated that CONUT score, PNI score, NLR, SII, SIRI, PIV, and MII-3 had discriminative value in differentiating diabetic (GDM and PGDM) from healthy pregnancies. Specifically, CONUT and PNI scores showed strong discriminative performance, with comparable trends also observed for systemic inflammatory indices (NLR, SII, SIRI) and composite indices (PIV, MII-3) in both diabetes groups (Figs. 1 and 2).

## Discussion

This study comprehensively evaluated the nutritional status and inflammatory indices of pregnant women diagnosed with gestational diabetes (GDM) and pregestational diabetes (PGDM) and compared them with healthy controls. Our findings have revealed significant immunometabolic differences between these groups, particularly showing that inflammatory activation and nutritional disorders are most pronounced in PGDM patients. This situation is also consistent with the literature highlighting the systemic effects of glucose irregularities during pregnancy. Unlike previous reports that mainly focused on either GDM or a limited number of biomarkers, our study integrates both nutritional (CONUT, PNI) and multiple inflammatory indices (NLR, SII, SIRI, PIV, MII-3) into a unified analytical framework.

This dual approach allowed a direct comparison between GDM and PGDM subtypes and revealed distinct immunometabolic profiles that may have practical implications for antenatal risk assessment.

Moreover, our study differs from earlier investigations not only by evaluating a broader biomarker panel but also by correlating these indices directly with adverse perinatal outcomes, such as preterm birth and NICU admission. This multidimensional design provides a novel perspective on the immunometabolic continuum between GDM and PGDM, which may inform early risk stratification and individualized management strategies.

It has emerged as a significant determinant in our analysis of nutritional status. The total CONUT score has shown moderate performance in distinguishing GDM from healthy controls (AUC = 0.659). Although the sensitivity in this cut-off is high (82.2%), the specificity is limited (47.3%). This finding suggests that the CONUT score may sensitively reflect nutritional stress, but its independent discriminative power may be limited. Importantly, this parameter should not be interpreted as a diagnostic tool for PGDM but rather as an adjunct for early risk stratification and prediction of adverse perinatal outcomes. These results align only partially with the findings of Tokgöz Çakır et al., who reported a superior discriminative performance of the PNI score in a cohort that excluded PGDM cases. This discrepancy may reflect the inclusion of PGDM patients in our study, who present with more pronounced metabolic and nutritional disturbances, thereby favoring the broader evaluative scope of the CONUT score [11].

In our analysis, the PNI score exhibited limited discriminatory capacity, with suboptimal performance in both the GDM (AUC = 0.382, *p* = 0.0438) and PGDM (AUC = 0.291) groups. Unlike the CONUT score—which incorporates serum albumin, total cholesterol, and lymphocyte count—the PNI is calculated using only albumin and lymphocyte parameters. This narrower composition may reduce its sensitivity to the multifaceted metabolic disturbances frequently observed in diabetic pregnancies.

A particularly noteworthy observation was the superior performance of the CONUT score in distinguishing PGDM cases from healthy controls (AUC = 0.787; sensitivity: 62.5%; specificity: 89.1%; threshold = 5.0). These findings suggest that the CONUT score may serve as a valuable tool for risk stratification in the preconceptional period, particularly among women with chronic metabolic and nutritional disorders. The clinical implications are significant, given that the PGDM group demonstrated both higher perinatal complication rates and more pronounced systemic inflammatory and nutritional disturbances.

Inflammatory markers also contributed meaningfully to our analysis. In the GDM group, both NLR and SII displayed modest yet statistically significant predictive value (AUC = 0.571 and 0.628, respectively). This is consistent with previous literature indicating that while these indices are not independently diagnostic, they do reflect the low-grade systemic inflammation commonly associated with GDM. Karataş et al. reported that first-trimester SII and AISI levels were associated with subsequent development of GDM and adverse neonatal outcomes, highlighting their potential as early predictive markers [12]. Similarly, Wang et al. observed elevated NLR, PLR, WBC, and neutrophil counts in GDM cases, suggesting their utility as accessible inflammatory biomarkers [13].

In the PGDM group, inflammatory indices demonstrated a more prominent predictive capacity. Notably, the SIRI and NLR markers exhibited moderate diagnostic performance, with AUC values of 0.679 and 0.667, respectively. These results are consistent with those reported by Xiu et al., who identified SII and SIRI as reliable indicators for predicting both maternal and neonatal complications in diabetic pregnancies [14]. Conversely, Hashemipour et al. reported that these derived inflammatory indices may not offer substantial prognostic advantage over conventional complete blood count parameters, suggesting that their incremental value remains debatable in certain clinical contexts [15]. This discrepancy may arise from patient selection in the studies, the threshold values used, or differences in population-based inflammatory responses.

From a metabolic standpoint, our findings are in agreement with those of Song et al., who reported that elevated triglyceride-glucose (TyG) index levels during the first trimester were associated with an increased risk of adverse pregnancy outcomes, including GDM, preeclampsia, and preterm birth [16]. They emphasized that early insulin resistance may contribute to the inflammatory sequelae of these complications, even in the absence of overt hyperglycemia. Similarly, our findings suggest that in cases of pregestational diabetes mellitus (PGDM), the combined burden of nutritional deficiency and systemic inflammation remains a key determinant of adverse perinatal outcomes.

Furthermore, the elevated neutrophil and total white blood cell (WBC) counts observed in both the GDM and PGDM groups in our study are consistent with the hematological profiles reported by Ye and Wang in their large-scale cohort analysis [13, 17]. These changes suggest that chronic and low-grade inflammation may interact with endothelial dysfunction and placental disorders, potentially leading to adverse pregnancy outcomes.

In the PGDM group, the increased rates of preterm birth, NICU admission, and Composite Adverse Perinatal Outcomes (CAPO) further underscore the contributory role of immune activation and nutritional compromise in obstetric morbidity. These findings are in line with the study by Mandic-Markovic et al., which demonstrated that fibrinogen levels and leukocyte-based inflammatory indices are associated with adverse neonatal outcomes in pregnancies complicated by GDM [18]. Abbasi Fashami and Li’s studies have similarly shown that inflammatory indices are significantly associated with poor glycemic parameters and pregnancy complications [7, 19].

One of the major strengths of our study is the separate and systematic evaluation of both gestational and pregestational diabetes groups, which allowed for a nuanced understanding of the immunometabolic differences between these subtypes. Unlike previous studies that have often grouped all diabetic pregnancies together, our design enabled a direct comparison of inflammatory and nutritional indices across two pathophysiologically distinct entities. This stratified approach provides clearer clinical insight into the severity and profile of systemic alterations in PGDM, which are often underrepresented in the literature. From a clinical perspective, the combined use of nutritional and inflammatory indices may serve as a practical and cost-effective tool to identify high-risk pregnancies before overt metabolic complications occur. Incorporating these indices into antenatal evaluation protocols could facilitate early dietary optimization and closer monitoring, particularly among women with pregestational diabetes. Furthermore, the incorporation of multiple validated indices—such as CONUT, PNI, NLR, SII, and SIRI—enabled a robust and multifaceted assessment of metabolic and inflammatory status. The use of ROC analysis further strengthened the objectivity of our findings by quantitatively determining the discriminative and prognostic capabilities of these biomarkers.

Conversely, this study has several limitations that warrant consideration. First, its retrospective design introduces inherent susceptibility to selection bias, limits control over confounding variables, and precludes the establishment of causal inferences. Second, the study was conducted in a single tertiary referral center, which may not reflect the broader population and limits external validity. Third, while we included a wide range of inflammatory and nutritional indices, the absence of serial measurements precluded assessment of temporal changes or response to clinical interventions, such as dietary modifications, insulin therapy, or glycemic control over time. Fourth, although the overall sample size was adequate for primary comparisons, subgroup analyses—especially those focusing on rare outcomes like stillbirth, severe neonatal morbidity, or specific CAPO components—may have been underpowered. Fifth, our study did not include detailed assessments of dietary intake, physical activity, or socioeconomic factors, which are known to influence both inflammatory status and nutritional profiles. Finally, the classification of PGDM was based on treatment history and available records, without further stratification into type 1 or type 2 diabetes, which may have masked subtype-specific variations in immunometabolic status.

## Conclusion

This study revealed distinct immunometabolic profiles among pregnant women with gestational and pregestational diabetes by comparatively evaluating their nutritional and inflammatory status. Notably, the PGDM group demonstrated markedly elevated total CONUT scores and systemic inflammatory indices, which were significantly associated with adverse perinatal outcomes, including preterm birth and NICU admission.

The high specificity of the CONUT score in identifying PGDM cases suggests its potential utility as a preconceptional risk stratification tool in women with chronic metabolic and nutritional disorders. In contrast, the limited discriminative ability of the PNI score underscores its inadequacy in capturing the multifactorial metabolic disturbances associated with diabetes.

Regarding inflammatory indices, elevated levels of SII, NLR, SIRI, and PIV in the PGDM group reflect a heightened chronic inflammatory state. ROC-based analyses revealed that these indices may hold promise for the early prediction of adverse perinatal outcomes, particularly in pregnancies complicated by PGDM.

Collectively, our findings suggest that both nutritional deficits and systemic inflammation contribute significantly to poor perinatal prognosis in diabetic pregnancies. Incorporating these parameters into antenatal risk assessment may enhance early identification of high-risk cases and inform targeted interventions. These findings further suggest that integrating CONUT and inflammatory indices into routine antenatal assessment may enhance individualized risk stratification and guide timely preventive interventions in clinical practice. Further large-scale, prospective, and longitudinal studies are warranted to validate these results and elucidate the temporal dynamics of these biomarkers across gestation.

## Data Availability

The datasets used and/or analyzed during the current study are available from the corresponding author on reasonable request.

## Abbreviations

GDM: Gestational Diabetes Mellitus
PGDM: Pregestational Diabetes Mellitus
CONUT: Controlling Nutritional Status
PNI: Prognostic Nutritional Index
NLR: Neutrophil-to-Lymphocyte Ratio
PLR: Platelet-to-Lymphocyte Ratio
MLR: Monocyte-to-Lymphocyte Ratio
SII: Systemic Immune-Inflammation Index
SIRI: Systemic Inflammation Response Index
PIV: Pan-Immune Inflammation Value
MII-1: Multi Inflammatory Index-1
MII-2: Multi Inflammatory Index-2
MII-3: Multi Inflammatory Index-3
CRP: C-Reactive Protein
BMI: Body Mass Index
FBG: Fasting Blood Glucose
WBC: White Blood Cell
NICU: Neonatal Intensive Care Unit
APGAR: Appearance, Pulse, Grimace, Activity, Respiration
CAPO: Composite Adverse Perinatal Outcome
OGTT: Oral Glucose Tolerance Test
ADA: American Diabetes Association
WHO: World Health Organization
AST: Aspartate Transaminase
ALT: Alanine Transaminase
TSH: Thyroid-Stimulating Hormone
HDL: High-Density Lipoprotein
LDL: Low-Density Lipoprotein
ROC: Receiver Operating Characteristic
AUC: Area Under the Curve
IQR: Interquartile Range
